# Oral findings in secondary syphilis

**DOI:** 10.4317/medoral.22196

**Published:** 2018-02-25

**Authors:** Rodrigo-Soares de Andrade, Edimilson-Martins de Freitas, Breno-Amaral Rocha, Edson-da Silva Gusmão, Mário-Rodrigues-Melo Filho, Hercílio-Martelli Júnior

**Affiliations:** 1 Department of Oral Diagnosis, School of Dentistry, State University of Campinas, FOP-UNICAMP, Piracicaba, São Paulo, Brazil; 2 Dentistry School, State University of Montes Claros, Unimontes, Minas Gerais State, Brazil; 3 Medicine School, State University of Montes Claros, Unimontes, Minas Gerais State, Brazil

## Abstract

**Background:**

Syphilis is a sexually transmitted disease caused by Treponema pallidum. However, there are of hematogenic and vertical transmission. All health care professionals must be aware of the manifestations of this condition, such as oral lesions.

**Objectives:**

This study to analyze and compare four clinical cases of syphilis that were diagnosed based on lesions in the oral cavity with published literature.

**Material and Methods:**

Four patients with a confirmed sorologic and clinical diagnosis of syphilis were examined, confirmated from manifestation of oral lesions together with analysis of serological laboratory tests and histopathological analyses.

**Results:**

Lesions were found in classic sites such as lips, tongue and skin. However, there were also lesions on the hard palate, and labial commissure, which correspond to less than 5% of the syphilis oral manifestations.

**Conclusions:**

The practice of unprotected oral sex may result in infection and development of syphilis. The acknowledgment of the oral manifestations of syphilis in all its period of training for health professionals is of basic importance, the association of clinical features, histopathological findings and serological tests are required to complete the diagnosis and correct treatment.

** Key words:**Oral syphilis, treponema infections, secondary syphilis.

## Introduction

Syphilis is a systemic bacterial infection caused by Treponema pallidum. It is estimated that there are more than 12 million cases per year in the world, out of which 900 thousand are in Brazil (1). The incubation period is usually 21 to 30 days after contact, although it can vary from 10 to 90 days, depending on the number and virulence of Treponemas and the host response (2).

Regarding the transmission pathways, the infection is mainly sexually transmitted, but it can also occur through hematological or vertical pathways. The signs and symptoms are different according to the disease stage (2). Oral manifestations are, in many cases, one of the first signs of the disease and can guide the correct and early diagnosis, which is of great importance for the treatment of this condition (1). The clinical features of syphilis are diverse, as in the case of recent primary infection characterized by cancrum and secondary mucocutaneous lesions with late infection represented by various signs and symptoms, such as vascular, and tegumentary, among others, and in the mouth the most important lesion is the syphilitic gum, a form of granulomatous inflammatory process of secondary state (2).

The patient affected with syphilis may present painless and non-inflammatory bilateral satellite adenopathy of submandibular and cervical lymph nodes (3). The lip represents the most common topography of involvement, followed by tongue and the tonsils (3,4). An important characteristic of the syphilitic lesion of the oral cavity is the absence of painful symptomatology; therefore, this condition must be differentiated from squamous cell carcinoma, a common malignant neoplasm in this anatomical region (3).

Secondary syphilis are headache, tearing, nasal secretion, pharyngitis, generalized arthralgia and myalgia (4). The disease, at this stage, is characterized by systemic involvement and diffuse and painless maculopapular cutaneous rash called syphilitic rosette (5). Sometimes, in addition to the cutaneous lesions, this systemic condition can be associated to palmoplantar lesions that can affect several areas of the oral cavity (5).

Clinically, in the oral cavity, oral chancre manifests as a self-limiting unspecific ulcer mostly affecting tongue, lips and commissure, characterizing primary syphilis (4). Secondary syphilis is associated with hematogenous dissemination of the microorganism and the clinical manifestations of the disease may be quite heterogeneous and unspecific (3-5). Witish/reddish oval macules covered by a fibrinous pseudomembrane or papular eruptions may be observed in the mucous membranes, associated or not with skin lesions (6). It may also present a form of condyloma latum, characterized by nodular, firm lesions or discretely elevated mucous plaques, which may be superficially eroded or ulcerated (7). Oral syphilis may eventually develop, manifestation as a nodular ulcerated granulomatous palatal mass or syphilitic glossitis (4). The objective of this study is to report four clinical cases of secundary syphilis that was diagnosed based on oral lesions.

## Material and Methods

Four patients diagnosed with syphilis from oral lesions were examined. All patients were recruited from the in the Oral Medicine Service of the State University of Montes Claros, Minas Gerais State, Brazil. In all cases, the syphilis was determined based on oral lesions, histopathologic and serologic exams. As inclusion criteria, patients with serological tests positive for syphilis with oral manifestations after oral sex practice were included. These patients should be HIV negative. The sex of the patients was not used as inclusion or exclusion factor in this study.

In addition, previously published data regarding oral manifestations of syphilis were analyzed. Literature reviews and clinical cases where the diagnosis of syphilis was initially addressed through oral lesions were used to compare the data with the clinical findings among the patients analyzed in this study.

All patients included in this study were submitted to a clinical evaluation, which included general and oral examinations. This study received approval from the Human Research Ethics Committee of the University. A signed statement of informed consent was obtained from all participants or their legal guardians.

## Results

**Figure 1:**
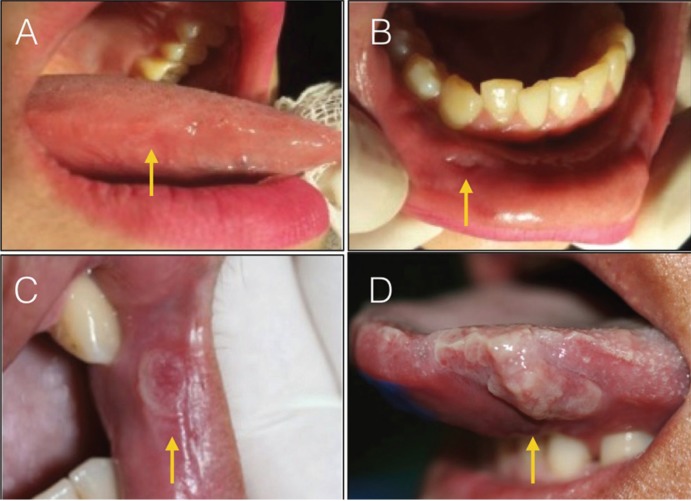
Different patterns of clinical presentation of secondary syphilis. (A) Grayish white lesion on lateral border of right tongue. (B) Circular ulcer and well delimited in asymptomatic lower labial mucosa (C) Ulcerated lesion with fibrinous borders at the commissure of the lips. (D) Exuberant mucous plate present on the tongue (left) caused volume increase and remodeling on the superficial relief of the tongue.

**Figure 2:**
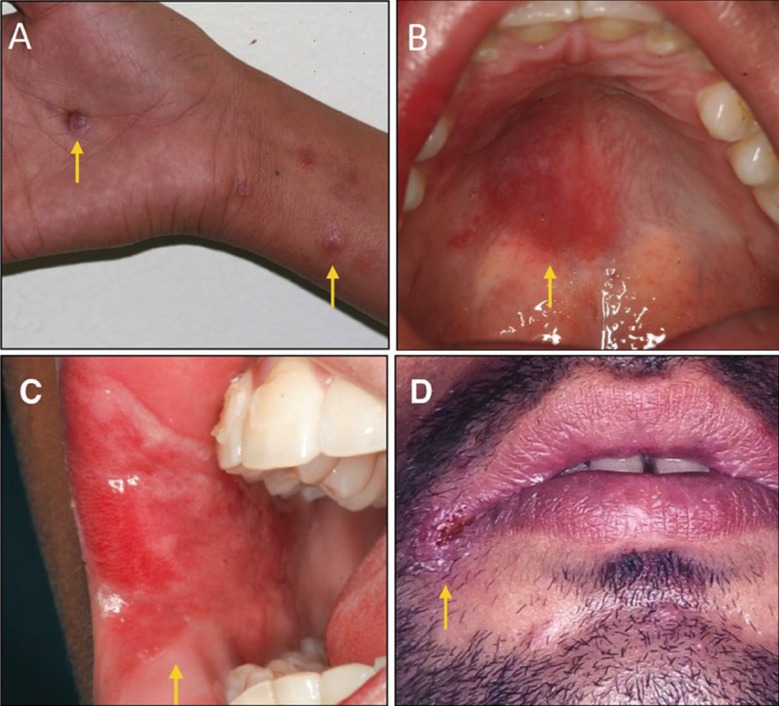
Oral and cutaneous clinical aspect of secondary syphilis (A) Reddish macules present in the palms of the hands and brackets. (B) Erythematous lesion present on the hard palate. (C) Extensive mucosal plaque present with erythematous areas in region of labial commissure and oral mucosa. (D) Ulcerated lesion present in labial commissure.

Four patients aged 17 to 42, of both sexes, were analyzed. Common and isolated symptoms were found between them ( Table 1). Patients were classified as 1 to 4 for better understanding in the study. Patients 1 and 2 presented bilaterally lesions on the tongue and lower lip (Fig. 1). Patients 3 and 4, in addition to oral lesions on lip and labial commissure, had blackened lesions on the skin and palms of the hands (Fig. 2).

**Table 1:**
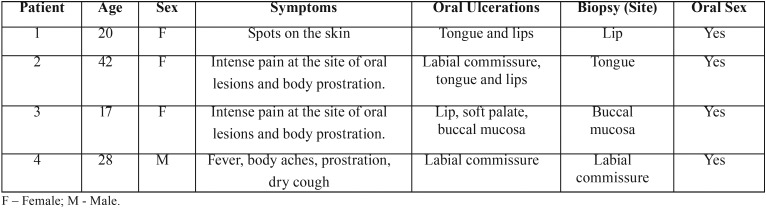
Clinical presentation of patients with secondary syphilis.

The patient 2, reported having used topical medications on his own, without improvement. The patient was related to the use a topical ointment based on neomycin sufate and hydrocortisone acetate, in order to heal the lesions. The patient 4 reported a lesion on the labial commissure without remission. In the first laboratory tests and incisional biopsy the results were inconclusive, only exarcebating the case. After the incisional biopsy, an acute inflammation reaction occured at the lesion site, causing edema and pain, a condition that was not found before the surgical procedure was performed. This made the patient felling more discomfort with the injury. After fifteen days, the exams were repeated, confirming the clinical diagnosis of secondary syphilis.

For all patients, specific laboratory tests for syphilis such as VDRL and FTA-ABS were requested, confirming positivity for syphilis.

**Figure 3:**
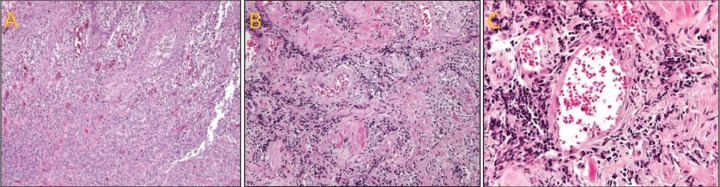
Histopathological aspects of oral syphilis. (A) Histopathological examination revealed hyperplasia of the epithelium. (B) A dense and diffuse chronic inflammatory infiltrate, composed mainly by lymphocytes and plasma cells, in the lamina propria. (C) The inflammatory infiltrate extended to the deeper area of the lamina propria and showed a perivascular pattern.

All patients in this study had a negative HIV test result. All the patients was submitted to an incisional biopsy on the tongue, labial commissure or lips, varying according to the location of the lesion of each patient, from which the histopathological examination revealed plasma cells, inflammatory infiltrate, presence of lymphocytes and plasma cells, endarteritis, and showed a perivascular pattern (Fig. 3). Thus, in the 4 reported cases of secondary syphilis, the diagnosis was established by the clinical, laboratory and microscopic characteristics.

## Discussion

The Treponema pallidum, the cause of syphilis, has humans as the only known host and cannot survive out of its natural host, for limited metabolic abilities, to synthesize its own bio nutrient (7). Syphilis is transmitted horizontally by sexual contact and vertically from mother to baby after 14 to 16 weeks gestation when Treponema pallidum crosses the placental barrier (7-9). Transmission of syphilis by means other than sexual contact has been reported in the literature, especially in children and health professionals. Although the genital areas are the most common site of manifestation, extra genital regions were cited for the onset of cancrum, such as oral cavity, fingers, nose, eyelids, arm and nipple (10), although they are unusual.

All patients had symptoms such as pharyngitis, myalgia, arthralgia, prostration, headache, generalized lymphadenopathy, characteristics of the secondary stage of syphilis. When they affect the oral cavity, they appear as oval reddish maculae or maculopapular eruptions (syphilitic rosette). In some situations, lesions similar to condyloma latum may occur in the oral cavity, with the labial commissure as the preferential site of involvement (11). Condyloma latum was observed in the second case presented in this study. This lesion is rarely reported (12). Patients observed to the study showed in the late moment of the disease, were the persistent ulcerations already installed. The presence of mucosal plaques, ulcerations in labial commissures and hard palate were observed, characterizing the secondary stage of the disease. The characteristic of lesion is a painless and heals spontaneously in 2 to 10 weeks, only 30 to 40% of patients are diagnosed at this time (13-14),

The cases presented in this study went through the primary infection without being diagnosed, where only after the development of painful ulcerations and skin patches, characteristic of the secondary stage of syphilis, could be correctly diagnosed.

The lesion may begin as a papule that may progress to a hard, painless, non-purulent, clean-based ulcer as mentioned in the cases presented in this study. The size of the lesions is variable and the margins are usually delimited. In the literature, the most affected anatomical sites are mainly the tongue, gingiva, soft palate and lips; in the cases presented, in addition to these sites, we had lesions present in the labial commissure and hard palate (15), in this study, mucosal plaque lesions in the tongue, lower lip, hard palate and labial commissure were observed, both the last two of which is rare.

In the secondary stage, which has more evident symptoms, the appearance of oral lesions is more common (16), occurring in all patients this study, 

probably by its route of infection. The mucous plaques are the most common clinical manifestation at this stage and are preferentially located on the lips, tongue, buccal mucosa and palate (17). At this stage, it is common the appearance of spots or mucous plaques slightly elevated and covered by white or grayish pseudo membranes. These lesions may also be associated with erythematous areas (18). Ulcerated lesions with irregular and whitish borders may also be observed. Red macules or erosive areas in the mucosa, nodular and maculopapular lesions may also occur (19). In cases reported in this study, the presence of mucous plaques was found in the oral cavity, associated with episodes of headache and myalgia. Due to the presence of oral manifestations, it was possible to suggest the diagnosis of secondary syphilis (20). The clinical presentation of oral secondary syphilis may be broad and varied, with several traumatic and infectious lesions as differential diagnosis. These include aphthous ulceration, oral ulceration associated with HIV, tuberculosis, lymphoma, mycosis, leshmaniosis, eosinophilic ulcer, squamous cell carcinoma, necrotizing sialometaplasia, cacroid, cytomegalovirus, gonorrhea and traumatic ulcers (21).

Patients 1 and 2 (Table 1) present lesions on the tongue and lips, which are usually described in the literature (9-15). The patient 3 and 4 (Table 1) case has several cutaneous lesions, all over the body and on the palms of the hands (pink or red symmetrical maculae, to the papular or pustular form). In the fourth case, the laboratory tests were initially inconclusive, but the ulceration at the labial commissure was still present, increasing the suspicions of syphilis. Only after the re-examination was it possible to confirm the positivity for syphilis, which was already clinically, clear. These sites of presentation of oral lesions when compared with data in the literature (10-19) are rare to be found. Because lesions in the lower lip and tongue are more commonly observed, and in patients 3 and 4 we present soft palate and lip commissure When we look at the sites of incidence of oral syphilis lesions we see that the most common sites are lips, tongue and tonsils (8) and when compared to the sites designated in this study, we observed rare sites affected, such as hard palate and labial commissure.

Oral manifestations are uncommon and may represent a diagnostic challenge because of their broad spectrum of clinical appearances (22). The characteristics of the disease may mimic other conditions and due to its increasing incidence in many parts of the world, it should be considered in the differential diagnosis of oral lesions (23).

Soares *et al.* drew attention to a probable increase in the incidence of oral manifestations of oral syphilis due to the increase in the practice of oral sex. However, the literature review shows that the number of case reports and citations in the literature regarding the occurrence of oral lesions does not seem to follow the recently reported “outbreaks” (24). According to Eyer-Silva *et al.* 2017 the occurrence of oral lesions in syphilis would be between 12-15%, while Lautenschlager, 2006, found oral manifestations in about one-third to one-half of patients with secondary syphilis (25,26).

Although there is no in literature studies direct correlation between syphilis and pratics of oral sex, we know that practice can transmit the Treponema pallidum, both through oral sex, through kissing in the mouth and sharing toothbrushes (27). In this study have a 100% frequency of appearance of syphilitic lesions in the mouth, after the practice of oral sex.

The histopathologic characteristics of secondary syphilis are as variable as the lesions. Whereas the changes are often nonspecific, findings of endothelial cell swelling, perivascular infiltrates with a preponderance of plasma cells, and epidermal psoriasiform hyperplasia support the diagnosis of syphilis (28). In reported cases in this study observed a hyperplasia of the epithelium, a dense and diffuse chronic inflammatory infiltrate, composed mainly by lymphocytes and plasma cells, in the lamina propria and the inflammatory infiltrate extended to the deeper area of the lamina propria and showed a perivascular pattern. In these patients, VDRL and FTA-ABS were chosen due to their low cost and degree of specificity (29). The histopathological examination was also carried out with the objective of discarding other possible diseases. Syphilis treatment consists of antibiotic therapy, is antibiotic to benzathine penicillin G (30) in which they were used in the treatment of patients of this study.

## Conclusions

The practice of unprotected oral sex may result in infection and development of syphilis. The acknowledgment of the oral manifestations of this disease by health professionals is of great importance, so that the diagnosis is made as early as possible An association of clinical features, histopathological findings and serological tests are required to complete the diagnosis of syphilis. Ulcerated lesions in lips are the most common oral manifestation found in patients with syphilis, the hard palate and labial commissure are the rare affected site.
